# The development and validation of oral cancer staging using administrative health data

**DOI:** 10.1186/1471-2407-14-380

**Published:** 2014-05-29

**Authors:** Chang Li-Ting, Chen Chung-Ho, Yang Yi-Hsin, Ho Pei-Shan

**Affiliations:** 1 Faculty of Dental Hygiene, College of Dental Medicine, Kaohsiung Medical University, Kaohsiung, Taiwan; 2 Kaohsiung Medical University Chung-Ho Memorial Hospital, Cancer Center, 100 Shih-Chuan First Rd, Kaohsiung 807, Taiwan; 3 Division of Oral and Maxillofacial Surgery, Department of Dentistry, Kaohsiung Medical University Hospital, Kaohsiung Taiwan; 4 School of Pharmacy, Kaohsiung Medical University, Kaohsiung, Taiwan

**Keywords:** Oral cancer, Validation, National health database, Taiwan

## Abstract

**Background:**

Oral cancer is a major global health problem. The complexity of histological prognosticators in oral cancer makes it difficult to compare the benefits of different treatment regimens. The Taiwanese National Health database provides an opportunity to assess correlations between outcome and treatment protocols and to compare the effects of different treatment regimens. However, the absence of indices of disease severity is a critical problem. The aim of this study was to ascertain how accurately we could assess the severity of oral cancer at the time of initial diagnosis on the basis of variables in a national database.

**Methods:**

In the cancer registry database of a medical center in Taiwan, we identified 1067 histologically confirmed cases of oral cancer (ICD9 codes 140, 141 and 143–145) that had been first diagnosed and subjected to initial treatment in this hospital. The clinical staging status was considered as the gold standard and we used concordance (C)-statistics to assess the model’s predictive performance. We added the predictors of treatment modality, cancer subsite, and age group to our models.

**Results:**

Our final overall model included treatment regimen, site, age, and two interaction terms; namely, interactions between treatment regimen and age and those between treatment regimen, site, and age. In this model, the C-statistics were 0.82–0.84 in male subjects and 0.96–0.99 in female subjects. Of the models stratified by age, the model that considered treatment regimen and site had the highest C-statistics for the interaction term, this value being greater than 0.80 in male subjects and 0.9 in female subjects.

**Conclusion:**

In this study, we found that adjusting for sex, age at first diagnosis, oral cancer subsite, and therapy regimen provided the best indicator of severity of oral cancer. Our findings provide a method for assessing cancer severity when information about staging is not available from a national health-related database.

## Background

Oral cancer is a major health problem, the worldwide annual incidence being 274,300 cases with 128,000 deaths; two-thirds of this burden is in developing countries [1]. Despite considerable advances in diagnostic and therapeutic techniques, oral cancer continues to portend a poor prognosis. We surveyed available published reports and found that the effect of treatment regimen or other prognosis-related factors is often uncertain and controversial [2-5]. The complexity of histological prognosticators in oral cancer likely partly accounts for this because it makes it difficult to compare the benefits of different treatment regimens; small samples are another limitation of previous studies [6-8].

The Taiwan National Health Insurance program, which has operated since 1995, enrolls almost 99% of the inhabitants of Taiwan and is contracted with 97% of hospitals and clinics throughout the nation [9]. It therefore provides an opportunity to assess correlations between outcome and treatment protocol and thus compare the effectiveness of different treatment regimens. However, the major purpose of this program concerns costs of medical services. In general, lack of information about disease severity is a critical problem when analyzing a population database. Anatomic site and disease stage are the most important tumor-related predictors of the prognosis of oral cancer after various treatment regimens [10-13]. The aim of this study was to try to assess how accurately the severity of oral cancer at the time of first diagnosis can be assessed on the basis of variables commonly available in national databases.

## Methods

### Database

We used data from a cancer registry database of a medical center in Taiwan. In our study, we included all patients with oral cancer (ICD9 codes 140, 141, 143–145) who had been first diagnosed and undergone initial treatment in this hospital from 1 January 2002 to 31 December 2007. All 1067 of the oral cancer subjects included in the database had been histologically confirmed and staged according to the TNM staging system of the Union for International Cancer Control [14]. Most study subjects had squamous cell carcinoma (SCC; 971 cases, 91%); 577 of these (54.08%) were well differentiated and 290 (27.18%) moderately differentiated. The Institutional Review Board of Kaohsiung Medical University Hospital reviewed and approved our proposal for use of the database (KMUH-IRB-980174).

Data concerning sex, age at first diagnosis, oral cancer subsite (lip, tongue, gum, floor of the mouth, and other sites), clinical stage, and therapy regimen were collected from the database. We considered seven different treatment regimens in this study; all were based on a combination of surgery, radiotherapy, and chemotherapy. The gold standard for classifying oral cancer is considered clinical stage, and we tried to classify it as accurately as possible by using available personal and medical intervention variables. We performed the χ^2^ test to ascertain which individual variables significantly contributed to the accuracy of staging. To assess the accuracy of our model’s predictive performance, we performed multivariate logistic regression analyses and used concordance (C) statistics. In the logistic regression analysis models, we included: (i) treatment modality (the categories were surgery only; radiation only; chemotherapy only; surgery and chemotherapy; surgery and radiation; radiation and chemotherapy; surgery, and radiation and chemotherapy; (ii) cancer subsite (lip [140], tongue [141], gum [143], floor of mouth [144], and other [145]); (iii) age group (20–44 years, 45–64 years and ≥65 years); and (iv) interactions of these treatments and sites.

A C-statistic of 1.0 represents perfect sensitivity and specificity; whereas a C-statistic of 0.5 represents an essentially worthless test. The C-statistic is an accuracy measure that can be used for ordinal or nominal outcomes. In this study, the C-statistic is a measure of the accuracy with which the model discriminates between patients who were diagnosed as early stage and those who were diagnosed as advanced stage.

## Results

More than 90% of our cases were male (995/1067). The mean first diagnosed age was 51.58 years (standard deviation (SD) = 11.12); 51.08 years (SD = 10.67) in male subjects and 58.64 years (SD = 14.44) in female subjects. More than 50% of all cases were in the age group of 45–65 years at the time of diagnosis; 60% of male subjects were in this age group. About 27% of male subjects were diagnosed before the age of 45 years, but only 15% of women. Relevant clinical variables at time of diagnosis are shown in Table 1. More than 50% of cases were first diagnosed at an advanced stage (III or IV), especially in men (>65%). Tongue and buccal mucosa were the dominant subsites of oral cancer in our study. About 30% of oral cancer in men originated in the tongue and 30% in the buccal mucosa; however, in women, the tongue (37.5%) was clearly the most common subsite. Surgery alone and chemotherapy alone were the two most commonly administered treatment regimens.

**Table 1:** Relevant clinical characteristics of patients with oral cancer

		**Male (n = 995)**		**Female (n = 72)**	
		**N**	**%**	**N**	**%**
Age					
	20-44	265	26.63	11	15.28
	45-65	610	61.31	32	44.44
	≧65	120	12.06	29	40.28
Stage					
	I	199	20.00	24	33.33
	II	137	13.77	4	5.56
	III	284	28.54	11	15.28
	IV	375	37.69	33	45.83
Site					
	Lip	48	4.82	4	5.56
	Tongue	307	30.85	27	37.50
	Gun	83	8.34	8	11.11
	Floor of mouth	26	2.61	4	5.56
	Palate	60	6.03	4	5.56
	Buccal	302	30.35	16	22.22
	Others and unspecified parts of mouth	169	16.98	9	12.50
Treatment					
	S alone	350	35.18	28	38.89
	RT alone	5	0.05	0	0.00
	CT alone	302	30.35	19	26.39
	S + RT	60	6.03	7	9.72
	S + CT	139	13.97	11	15.28
	RT + CT	69	6.93	4	5.56
	S + RT + CT	70	7.04	3	4.17

S: surgery; R: radiation; C: chemotherapy.

Tables 2 and 3 show the distribution of relevant factors in each sex according to clinical stage. In male patients, age, site, and treatment regimens were significantly associated with clinical stage (stage I vs II–IV and clinical stage I–II vs III–IV). However, for clinical stages I–III versus IV, age was not a significant factor, whereas site and treatment were. In female patients, age was not a significant factor for any of these comparisons. Site was the only factor that was statistically significantly associated with all comparison situations. The factor of treatment regimen showed different patterns of association for different staging combinations; however, none of these were statistically significant because there too few cases in any one category of treatment regimen. Tables 4 and 5 show the stepwise logistic regression models with which we examined the accuracy of the different predictors. In Model 1 of Table 4, only treatment regimens are considered; the C-statistics are all 0.76 for the various combinations compared in male subjects and 0.83–0.85 in female subjects. Model 2 included only site; the C-statistics are 0.60–0.64 in male patients and 0.77–0.82 in female patients. Model 3 included treatment regimen and site; the C-statistics are 0.78–0.79 in male subjects and 0.91–0.96 in female subjects. Interactions between treatment regimens and sites are considered in Model 4; the C-statistics are 0.79–0.81 in male patients and 0.94–0.97 in female patients. Following Model 4, age was considered in Model 5; the C-statistics are 0.80–0.82 in male subjects and 0.96-0.99 in female subjects. The final model shown is Model 6, which included treatment regimen, site, age and two interaction terms; namely, the interaction effect of treatment regimen/age and of treatment regimen/site/age. The C-statistics in Model 6 are 0.82–0.84 in male patients and 0.96–0.99 in female patients. In Table 5, the models are stratified by age and the accuracy evaluated by the predictors of treatment regimen and site. There are four models in this table; these consider treatment regimen, site, treatment regimen, and site, and adding the interaction terms of the two factors in each of Models 1, 2, 3, and 4 separately. For each stratified group, Model 4 has the highest C-statistics, the values being greater than 0.80 in male patients and 0.9 in female patients. The accuracy tended to be better in older age groups, but we found no significant variations in the various age groups.

**Table 2:** Distribution of relevant factors in male patients according to clinical stage

	**Male**										
			**Stage I versus II-IV**			**Stage I-II versus III-IV**			**Stage I-III versus IV**		
			**Stage I**			**Stage I-II**			**Stage I-III**		
		**Total**	**N**	**%**	**P-value**	**N**	**%**	**P-value**	**N**	**%**	**P-value**
Age					0.0056			0.0423			0.9638
	20-44	265	42	15.85		78	29.43		164	61.89	
	45-64	610	121	19.84		207	33.93		380	62.30	
	> = 65	120	36	30.00		51	42.50		76	63.33	
Site					<.0001			<.0001			<.0001
	Lip	48	13	27.08		19	39.58		38	79.17	
	Tongue	307	84	27.36		133	43.32		233	75.90	
	Gingiva	83	5	6.02		13	15.66		25	30.12	
	Floor of mouth	26	11	42.31		15	57.69		19	73.08	
	Others	531	86	16.20		156	29.38		305	57.44	
Treatment					<.0001			<.0001			<.0001
	S	346	142	40.57		215	61.43		305	87.14	
	RT	5	2	40.00		2	40.00		2	40.00	
	CT	302	18	5.96		38	12.58		109	36.09	
	S + RT	60	11	18.33		18	30.00		43	71.67	
	S + CT	139	18	12.95		43	30.94		92	66.19	
	RT + CT	69	0	0.00		4	5.80		23	33.33	
	S + RT + CT	70	8	11.43		16	22.86		46	65.71	

S: surgery; R: radiation; C: chemotherapy.

**Table 3:** Distribution of relevant factors in female patients according to clinical stage

**Female**										
		**Stage I versus II-IV**			**Stage I-II versus III-IV**			**Stage I-III versus IV**		
		**Stage I**			**Stage I-II**			**Stage I-III**		
		**N**	**%**	**P-value**	**N**	**%**	**P-value**	**N**	**%**	**P-value**
Age				0.2566			0.4916			0.4026
	20-44	6	54.55		6	54.55		8	72.73	
	45-64	10	31.25		11	34.38		16	50.00	
	> = 65	8	27.59		11	37.93		15	51.72	
Site				0.0015			<.0001			0.0005
	Lip	2	50.00		3	75.00		3	75.00	
	Tongue	16	59.26		19	70.37		23	85.19	
	Gingiva	1	12.50		1	12.50		2	25.00	
	Floor of mouth	2	50.00		2	50.00		2	50.00	
	Others	3	10.34		3	10.34		9	31.03	
Treatment										
	S	17	60.71		20	71.43		24	85.71	
	RT	0	0.00		0	0.00		0	0.00	
	CT	0	0.00		0	0.00		3	15.79	
	S + RT	4	57.14		4	57.14		6	85.71	
	S + CT	2	18.18		3	27.27		5	45.45	
	RT + CT	1	25.00		1	25.00		1	25.00	
	S + RT + CT	0	0.00		0	0.00		0	0.00	

S: surgery; R: radiation; C: chemotherapy.

**Table 4:** Staging accuracy according to logistic regression models evaluating the variables of treatment, site, and age

	**Model 1**	**Model 2**	**Model 3**	**Model 4**	**Model 5**	**Model 6**
**Variables**	**Treatment**		**Treatment**	**Treatment**	**Treatment**	**Treatment**
**In**		**Site**	**Site**	**Site**	**Site**	**Site**
**Models**					**Age**	**Age**
				Treatment*site	Treatment*site	Treatment*site
						Treatment*site*age
Outcome (subgroups)	C-statistic					
Stage I versus II-IV						
Male	0.76	0.62	0.79	0.81	0.82	0.84
Female	0.83	0.77	0.91	0.94	0.96	0.97
Stage I-II versus III-IV						
Male	0.76	0.60	0.78	0.79	0.80	0.82
Female	0.85	0.82	0.96	0.97	0.99	0.99
Stage I-III versus IV						
Male	0.76	0.64	0.79	0.80	0.81	0.82
Female	0.85	0.78	0.93	0.94	0.96	0.96

**Table 5:** Accuracy of each model according to logistic regression analysis of various combinations of predictors

	**Model 1**	**Model 2**	**Model 3**	**Model 4**
Treatment	ˇ		ˇ	ˇ
Site		ˇ	ˇ	ˇ
Treatment*site				ˇ
C-statistic				
Male				
Stage I versus II-IV				
20-44	0.76	0.60	0.78	0.83
45-64	0.78	0.62	0.80	0.82
> = 65	0.77	0.68	0.81	0.86
Stage I-II versus III-IV				
20-44	0.75	0.63	0.78	0.83
45-64	0.77	0.58	0.78	0.79
> = 65	0.81	0.68	0.86	0.90
Stage I-III versus IV				
20-44	0.75	0.61	0.79	0.81
45-64	0.77	0.64	0.79	0.81
> = 65	0.76	0.73	0.86	0.89
Female				
Stage I versus II-IV				
20-44	0.93	0.70	1.00	1.00
45-64	0.87	0.82	0.89	0.92
> = 65	0.88	0.92	0.98	0.98
Stage I-II versus III-IV				
20-44	0.93	0.70	1.00	1.00
45-64	0.90	0.85	0.93	0.95
> = 65	0.87	0.97	1.00	1.00
Stage I-III versus IV				
20-44	0.98	0.77	0.98	0.98
45-64	0.91	0.87	0.96	0.96
> = 65	0.84	0.86	0.92	0.94

## Discussion

Knowledge of the anatomy and disease staging is essential to optimal treatment planning [15]. Some anatomic sites, such as the superior gingivolabial sulcus, are linked with poor outcomes because of their rich lymphatic drainage and difficulty in evaluating the extent of local invasion, and therefore in selecting an appropriate management strategy [16]. Vascular and lymphatic networks, which vary between different anatomic sites, may influence tumor evolution and hence the outcome; thus, SCCs at the base rather than the oral part of the tongue have a higher rate of metastasis [17]. Cancer staging reflects both homogeneous survival data and important variations in disease characteristics that affect treatment options. Differentiation between stages I or II and stages III or IV of oral SCCs is most important for treatment planning, because early-stage tumors (stages I and II) typically require only single-modality therapy (mostly surgical resection), whereas stage III and IV tumors may require multimodality therapy with a combination of chemotherapy, radiation, and surgical resection. The appropriate therapeutic modalities depend on the site of origin of the primary tumor [18]. Population-based administrative data are an effective source of information about chronic disease or for cancer surveillance. However, the ways in which data can be extracted from such databases differ; in practice certain categories of clinical information may be unavailable.

This study provides a method for adjusting for cancer severity when staging information is not available. We found that the severity of oral cancer can be assessed based on sex, age at first diagnosis, oral cancer subsite, and therapy regimen with an accuracy of 84% in male subjects and more than 96% in female subjects. In Taiwan, oral cancer is a male-dominant cancer, the male:female ratio being 9:1 [19]. More than 70% of men with oral cancer have the habits of both chewing and smoking tobacco, whereas only approximately 10% of female patients have these habits [20]. Although some studies have failed to find an association between prognosis and smoking tobacco or consuming alcohol [21], most authors have reported higher mortality in smokers and alcohol drinkers [22,23]. In a study from Taiwan [21], Lo *et al*. reported that areca quid chewing is also correlated with a poor prognosis. Smokers and alcohol drinkers seem to be at higher risk of developing second primary oral cancers than nonsmokers and nondrinkers; thus, they face worse outcomes [24,25]. In our study, we found that the sex of the patient seemed to affect the choice of treatment plan: a higher proportion of male than female patients had undergone combined multimodality therapy, especially those with early-stage disease. This finding may be related to the sexes having different habits; it requires further study.

Previous studies have suggested that sex differences in oral cancer prognosis are attributable to a delay in seeking medical care and differences in rate of compliance with recommended treatment. Some studies have reported lower survival rates in female subjects [22,26], whereas others have found no sex-based difference in prognosis [21,27,28]. A correlation between prognosis and age is controversial; some authors reporting they are unrelated and others having found that older patients have worse prognoses [22,23]. Most researchers accept that disease staging has a crucial influence on outcome [21,28-30].

This study has some limitations. Patients were included on the basis of a previous diagnosis of oral cancer. The training and expertise of the personnel who performed the pathological assessments is unknown; therefore, we are unable to determine the reliability of their findings. Measurement methods and diagnostic criteria were also likely variable. However, because the database used was from a medical center, its accuracy is reliable.

## Conclusion

The main conclusion of this study is that adjusting for sex, first diagnosed age, oral cancer subsite, and therapy regime facilitates accurate assessment of the severity of oral cancer. Our findings provide a method for adjusting for cancer severity when staging information is not available from national health-related databases.

## Competing interests

The authors declare that they have no competing interests.

## Authors’ contributions

PSH and LTC designed, conducted, and implemented the study and drafted the manuscript. CHC critically revised the original draft of the manuscript. YYH conceived the study, participated in its design and coordination, and helped to draft the manuscript. All authors have read and approved the final manuscript.

## Pre-publication history

The pre-publication history for this paper can be accessed here:

http://www.biomedcentral.com/1471-2407/14/380/prepub
